# The phosphodiesterase 10 inhibitor papaverine exerts anti-inflammatory and neuroprotective effects via the PKA signaling pathway in neuroinflammation and Parkinson’s disease mouse models

**DOI:** 10.1186/s12974-019-1649-3

**Published:** 2019-12-02

**Authors:** Yu-Young Lee, Jin-Sun Park, Yea-Hyun Leem, Jung-Eun Park, Do-Yeon Kim, Youn-Hee Choi, Eun-Mi Park, Jihee Lee Kang, Hee-Sun Kim

**Affiliations:** 1 Department of Molecular Medicine and Tissue Injury Defense Research Center, School of Medicine, Ewha Womans University, 808-1 Magok-dong, Gangseo-gu, Seoul, 07804 South Korea; 2 Department of Physiology and Tissue Injury Defense Research Center, School of Medicine, Ewha Womans University, Seoul, South Korea; 3 Department of Pharmacology and Tissue Injury Defense Research Center, School of Medicine, Ewha Womans University, Seoul, South Korea; 4 Department of Brain & Cognitive Sciences, Ewha Womans University, Seoul, South Korea

**Keywords:** Parkinson’s disease, Neuroinflammation, Neuroprotection, Microglia, PDE10 inhibitor, Papaverine, PKA signaling

## Abstract

**Background:**

Neuroinflammation plays a pivotal role in the pathogenesis of Parkinson’s disease (PD). Thus, the development of agents that can control neuroinflammation has been suggested as a promising therapeutic strategy for PD. In the present study, we investigated whether the phosphodiesterase (PDE) 10 inhibitor has anti-inflammatory and neuroprotective effects in neuroinflammation and PD mouse models.

**Methods:**

Papaverine (PAP) was utilized as a selective inhibitor of PDE10. The effects of PAP on the expression of pro-inflammatory molecules were examined in lipopolysaccharide (LPS)–stimulated BV2 microglial cells by ELISA, RT-PCR, and Western blot analysis. The effects of PAP on transcription factors were analyzed by the electrophoretic mobility shift assay, the reporter gene assay, and Western blot analysis. Microglial activation and the expression of proinflammatory molecules were measured in the LPS- or MPTP-injected mouse brains by immunohistochemistry and RT-PCR analysis. The effect of PAP on dopaminergic neuronal cell death and neurotrophic factors were determined by immunohistochemistry and Western blot analysis. To assess mouse locomotor activity, rotarod and pole tests were performed in MPTP-injected mice.

**Results:**

PAP inhibited the production of nitric oxide and proinflammatory cytokines in LPS-stimulated microglia by modulating various inflammatory signals. In addition, PAP elevated intracellular cAMP levels and CREB phosphorylation. Treatment with H89, a PKA inhibitor, reversed the anti-inflammatory effects of PAP, suggesting the critical role of PKA signaling in the anti-inflammatory effects of PAP. We verified the anti-inflammatory effects of PAP in the brains of mice with LPS-induced systemic inflammation. PAP suppressed microglial activation and proinflammatory gene expression in the brains of these mice, and these effects were reversed by H89 treatment. We further examined the effects of PAP on MPTP-injected PD model mice. MPTP-induced dopaminergic neuronal cell death and impaired locomotor activity were recovered by PAP. In addition, PAP suppressed microglial activation and proinflammatory mediators in the brains of MPTP-injected mice.

**Conclusions:**

PAP has strong anti-inflammatory and neuroprotective effects and thus may be a potential candidate for treating neuroinflammatory disorders such as PD.

## Background

Neuroinflammation plays an important role in the development and progression of neurodegenerative diseases such as Parkinson’s disease (PD), Alzheimer’s disease, and Huntington’s disease (HD) [1, 2]. Microglia are the resident immune cells of the brain, and play a pivotal role in neuroinflammation. Microglia are readily activated following brain injury, during neurodegenerative processes, or upon interaction with misfolded proteins or invading pathogens. These cells then quickly proliferate, become hypertrophic, and secrete proinflammatory cytokines and neurotoxic factors [3, 4]. A prolonged and unresolved inflammatory response leads to destructive chronic inflammation (neuroinflammation), which results in neuronal cell death and the onset of neurodegenerative diseases [5, 6]. PD is characterized by impairment of the nigrostriatal dopaminergic system, which involves a severe loss of nigral neurons and a decrease in striatal dopaminergic input. In addition, the intracellular inclusion of α-synuclein is closely related to sustained neuroinflammation [7, 8]. The levels of pro-inflammatory cytokines are increased in the brain and cerebrospinal fluid of PD patients [9]. Thus, controlling microglial activation has been suggested as an important therapeutic strategy for neurodegenerative diseases.

Cyclic AMP (cAMP; 3′,5′-adenosine cyclic monophosphate), a second messenger in intracellular signaling, is known to regulate microglial function and activation [10, 11]. Moreover, recent studies have reported that cAMP is a critical determinant in the M1-M2 polarization of microglia [12, 13]. Intracellular cAMP level is controlled by phosphodiesterases (PDEs), enzymes that catalyze the hydrolysis of phosphodiester bonds and convert cyclic nucleotides into non-cyclic forms. There exist several reports demonstrating that PDE inhibitors exert anti-inflammatory effects in neuroinflammatory conditions. The PDE4 inhibitor FFPM improved learning and memory in APP/PS1 transgenic mice via its anti-inflammatory effect mediated through PKA/CREB signaling [14]. In addition, ibudilast, a non-selective PDE inhibitor, attenuated neuroinflammation by inhibiting astroglial reactivity in a MPTP mouse model of PD [15]. Until now, studies into the suppression of neuroinflammation by PDE inhibitors have mainly focused on PDE4, a predominant negative regulator of cAMP signaling within microglia [7]. Although the most common PDE4 inhibitor, rolipram, reached clinical trials, it later failed due to significant side effects [16, 17]. More recently developed inhibitors are also limited in their therapeutic role due to a lack of brain penetration [13]. Therefore, the development of a blood-brain barrier (BBB)-permeable PDE inhibitor with minimal side effects is necessary.

Papaverine (PAP; 6,7-dimethoxy-1-veratryl-isoquinoline) is a non-narcotic opium alkaloid isolated from *Papaver somniferum*, the opium poppy [18]. PAP is used clinically as a vasodilator and smooth muscle relaxant in conditions associated with spasm and dysmotility of the gastrointestinal and urinary tracts [19, 20]. PAP is a selective inhibitor of PDE10A, which is markedly expressed in the striatum of the brain [21, 22], specifically in the caudate nucleus, nucleus accumbens, and olfactory tubercle [23]. In addition, PDE10A has also been detected in parenchymal parts of the substantia nigra, globus pallidus, and striatonigral projections [24]. Therefore, several studies have suggested PDE10A as a potential therapeutic target for movement disorders such as PD and HD, and psychiatric disorders that affect the basal ganglia [25–27]. Currently, several PDE10A inhibitors are undergoing clinical trials in patients with HD and schizophrenia [28, 29]. Several studies have already identified therapeutic effects of PAP. PAP improved cognitive impairment in a HD mouse model by increasing GluA1 and CREB phosphorylation [30]. Moreover, PAP showed anti-inflammatory effects in mouse models of optic neuropathy, LPS-stimulated macrophages/microglia, and high mobility group box 1-mediated inflammatory responses [31–34].

Although previous studies have reported the various pharmacological activities of PAP, its effects on neuroinflammation and neurodegeneration have not been clearly demonstrated. Therefore, in the present study, we investigated whether PAP has therapeutic effects in neuroinflammation and PD mouse models. We found that PAP has anti-inflammatory effects in LPS-induced neuroinflammatory conditions, both in vitro and in vivo. We also demonstrated the neuroprotective and anti-inflammatory effects of PAP in MPTP-induced PD model mice. Further mechanistic studies revealed that the anti-inflammatory and neuroprotective effects of PAP are mediated through the PKA signaling pathway.

## Methods

### Animals

Adult male ICR (28–32 g, 8 weeks old) and C57BL/6 (22–25 g, 8 weeks old) mice were purchased from Orient Bio Inc. (Seongnam, Korea), a branch of Charles River Laboratories. Mice were housed at 21 °C under a 12 h light:12 h dark cycle, and had ad libitum access to water and rodent chow. Every effort was made to minimize stress to the animals. All experiments were performed in accordance with the National Institutes for Health and Ewha Womans University guidelines for Laboratory Animals Care and Use, and the study was approved by the Institutional Animal Care and Use Committee of the Medical School of Ewha Womans University (#ESM 15-0321).

### Reagents and antibodies

LPS (Escherichia coli serotype 055:B5), papaverine, H89, and antibodies against BDNF, iNOS, and β-actin were obtained from Sigma-Aldrich (St Louis, MO, USA). MPTP was purchased from Tokyo Chemical Industry Co. (Tokyo, Japan). Antibodies against phosphorylated and total Akt, MAPKs, and CREB were purchased from Cell Signaling Technology (Beverley, CA, USA). An antibody against phospho-p47phox was purchased from Assaybiotech (Sunnyvale, CA, USA). Antibodies against TNF-α, IL-1β, CD11b, lamin A, PDE10, and TH were purchased from Santa Cruz Biotechnology (Santa Cruz, CA, USA). Antibodies against IL-10, Bcl2, PGC-1α, and GDNF were purchased from Abcam (Cambridge, UK). An antibody against Iba-1 was purchased from Wako (Osaka, Japan). All RT-PCR enzymes and chemicals and electrophoretic mobility shift assay (EMSA) oligonucleotides were purchased from Promega (Madison, WI, USA). All other chemicals were obtained from Sigma-Aldrich, unless otherwise stated.

### Microglial cell culture and cell viability test

The immortalized mouse BV2 microglial cell line [35] was grown and maintained in Dulbecco’s modified Eagle medium supplemented with 10% heat-inactivated fetal bovine serum, streptomycin (10 μg/mL), and penicillin (10 U/mL) at 37 °C under 5% CO_2_. Primary microglial cells were cultured from the cerebral cortices of 1- to 2-day-old Sprague-Dawley rat pups as described previously [36]. The purity of the microglial cultures was > 95%, as confirmed by Western blot and immunocytochemistry analyses using an antibody specific to ionized calcium binding adapter protein-1 (Iba-1; data not shown). Cell viability was determined using the 3-(4,5-dimethylthiazol-2-yl)-2,5-diphenyl tetrazolium bromide (MTT) reduction assay, as previously described [37].

### Measurement of cytokine, nitrite, intracellular ROS, and cAMP levels in BV2 cells

BV2 cells (1 × 10^5^ cells per well in a 24-well plate) were pretreated with PAP 1 h prior to LPS stimulation (100 ng/mL) for 16 h. The supernatants of the cultured cells were collected and the concentrations of TNF-α, IL-1β, and IL-10 were measured by an enzyme-linked immunosorbent assay (ELISA) with a kit supplied from BD Biosciences (San Jose, CA, USA). Accumulated nitrite levels were measured using Griess reagent (Promega, Madison, WI). The intracellular accumulation of ROS was measured with H_2_DCF-DA (Sigma-Aldrich, St. Louis, MO), as previously described [38]. To determine the effect of PAP on intracellular cAMP level, BV2 cells were treated with PAP with or without LPS stimulation for 30 min, and cAMP level was measured using a cAMP ELISA kit from Enzo Life Sciences (Farmingdale, NY, USA).

### Reverse-transcription polymerase chain reaction

Total RNA from BV2 cells and mouse brain tissue was extracted using TRIzol reagent (Invitrogen, CA, USA). For RT-PCR, 1 μg RNA was reverse transcribed in a reaction mixture containing 1 U RNase inhibitor, 500 ng random primer, 3 mM MgCl_2_, 0.5 mM dNTP, 1X RT buffer, and 10 U reverse transcriptase (Promega, Madison, WI). The synthesized cDNA was used as a template for the PCR reaction using Go Taq polymerase (Promega) and primers for target genes. RT-PCR was carried out in a Bio-Rad T100 thermal cycler (Bio-Rad, Richmond, CA). Quantitative RT-PCR was performed on an ABI PRISM 7000 Sequence Detection System (Applied Biosystems, Foster City, CA) with Sensi FAST™ SYBR Hi-ROX Mix (Bioline, London, UK). The expression levels of the target genes were normalized against that of GADPH using the following formula: 2^(Ct, test gene - Ct, GAPDH)^. The primer sequences used in the PCR reactions are shown in Table 1.

**Table 1:** Primer sequences used for PCR

Gene	Forward primer (5′→3′)	Reverse primer (5′→3′)	Size
TNF-α	CCTATGTCTCAGCCTCTTCT	CCTGGTATGAGATAGCAAAT	354 bp
iNOS	CAAGAGTTTGACCAGAGGACC	TGGAACCACTCGTACTTGGGA	450 bp
IL-1β	GGCAACTGTTCCTGAACTCAACTG	CCATTGAGGTGGAGAGCTTTCAGC	447 bp
IL-6	CACGATTTCCCAGAGAACATGTG	ACAACCACGGCCTTCCCTACTT	128 bp
IL-10	GCCAGTACAGCCGGGAAGACAATA	GCCTTGTAGACACCTTGGTCTT	409 bp
MMP-3	ATTCAGTCCCTCTATGGA	CTCCAGTATTTGTCCTCTAC	375 bp
MMP-8	CCAAGGAGTGTCCAAGCCAT	CCTGCAGGAAAACTGCATCG	180 bp
p47phox	CGATGGATTGTCCTTTGTGC	ATCACCGGCTATTTCCCATC	256 bp
p67phox	CCCTTGGTGGAAGTCCAAAT	ATCCTGGATTCCCATCTCCA	242 bp
gp91phox	ACTGCGGAGAGTTTGGAAGA	GGTGATGACCACCTTTTGCT	201 bp
p22phox	AAAGAGGAAAAAGGGGTCCA	TAGGCTCAATGGGAGTCCAC	239 bp
TLR2	GAGCGAGCTGGGTAAAGTAG	AGCCGAGGCAAGAACAAAGA	528 bp
TLR4	GAGATGAATACCTCCTTAGTGTTGG	ATTCAAAGATACACCAACGGCTCTGA	414 bp
GAPDH	TTCACCACCATGGAGAAGGC	GGCATGGACTGTGGTCATGA	245 bp

### Electrophoretic mobility shift assay

BV2 cells were pretreated with PAP for 1 h and stimulated with LPS for 3 h. The nuclear extracts from the cells were prepared as previously described [36]. Double-stranded DNA oligonucleotides containing the NF-κB, AP-1, ARE, and CRE consensus sequences were end labeled using T4 polynucleotide kinase (New England Biolabs, Beverly, MA) in the presence of [γ-^32^P]ATP. Nuclear proteins (5 μg) were then incubated with a ^32^P-labeled probe on ice for 30 min, resolved on a 5 % acrylamide gel, and visualized by autoradiography.

### Western blot analysis

Whole cell protein lysates and brain tissue homogenates were prepared in a lysis buffer (10 mM Tris (pH 7.4), 30 mM NaCl, 1% Triton X-100, 0.1% SDS, 0.1% sodium deoxycholate, and 1 mM EDTA) containing a protease inhibitor cocktail. Protein samples were separated by SDS-PAGE, transferred to a nitrocellulose membrane, and incubated with primary antibodies according to the manufactures’ directions for dilution (Additional file 1: Table S1). After thoroughly washing with TBST, HRP-conjugated secondary antibodies (BioRad, Hercules, CA, USA 1:1000 dilution in TBST) were applied, and the blots were developed using an enhanced chemiluminescence detection kit (Thermo Fisher Scientific, Waltham, MA, USA). For quantification, the density of specific target bands was normalized against β-actin, using ImageJ software, version 1.37 (National Institutes of Health, Bethesda, MD).

### Transient transfection and luciferase assay

BV2 cells (2 × 10^5^ cells/well on a 12-well plate) were transfected with 1 μg of reported plasmid DNA using Metafectene transfection reagent (Biontex, Martinsried/Planegg, Germany). The effect of PAP on reporter gene activity was determined by pre-treating the cells with PAP prior to stimulation with LPS (100 ng/mL) for 6 h. After preparing the cell lysates, the luciferase assay was performed as previously described [36].

### Drug administration

ICR mice were randomly divided into six groups (control, LPS, LPS+PAP, LPS+PAP+H89, PAP, and H89; each group, *n* = 8–10). H89 (1 mg/kg, i.p.) was administrated 1 h before PAP (30 mg/kg/day, i.p.) for four consecutive days. A single injection of LPS (5 mg/kg, i.p.) was administered 1 h after the final PAP administration as previously described [36]. For studying the MPTP mouse model, C57/BL6 mice were divided into six groups (control, MPTP, MPTP+PAP, MPTP+PAP+H89, PAP, and H89; each group, *n* = 12–14). H89 (1 mg/kg, i.p.) was administrated 1 h before PAP (30 mg/kg/day, i.p.) for three consecutive days. One day after the final PAP treatment, MPTP (20 mg/kg, i.p) was injected four times with 2-h intervals [39].

### Behavioral test

To assess mouse motor coordination, the rotarod test (20–21 rpm, 600 s), modified from a previous method [40], was performed 1, 3, and 7 days after MPTP injection. Before the principal test, all mice were trained on the rotarod (18–19 rpm) until no fall was observed in 300 s. To evaluate dyskinesia, the pole test (50 cm in height, 0.7 cm in diameter, 120 s) was implemented 6 days after MPTP injection. Similarly, prior to the principal test, mice were trained three times to successfully descend from the top to the bottom of the pole.

### Brain tissue preparation

For histological analysis, the mice were anesthetized with sodium pentobarbital (80 mg/kg body weight, i.p. injection) and were then transcardially perfused with 0.9% saline followed by 4% paraformaldehyde for tissue fixation. The brains were then isolated and stored in 30% sucrose solution at 4 °C for cryoprotection. For biochemical analysis, the mice were transcardially perfused with saline. The striatum and substantia nigra were dissected from each brain according to the Paxinos mouse brain atlas, and immediately frozen in liquid nitrogen until use.

### Immunohistochemistry and immunofluorescence analysis

Using a cryotome (CM1860; Leica, Mannheim, Germany), 40-μm-thick coronal sections were cut, and were then stored in anti-freezing solution (30% ethylene glycol and 30% glycerol in phosphate-buffered saline) at − 20 °C. For immunohistochemical (IHC) staining, sections were treated with 3% H_2_O_2_ and 4% BSA to inactivate endogenous peroxidation and block non-specific binding, respectively. Sections were incubated with primary antibodies overnight and incubated with biotinylated secondary antibodies for 1 h at 25 °C room temperature, followed by an avidin-biotin-HRP complex reagent solution (Vector Laboratories, Burlingame, CA, USA). Subsequently, the peroxidase reaction was performed using diaminobenzidine tetrahydrochloride (Vector Laboratories). For double immunofluorescence (IF) staining, sections were treated to block non-specific binding and were incubated with primary antibodies, followed by secondary antibodies conjugated to a fluorochrome. Detailed information on the primary antibodies used is presented in Additional file 1: Table S2. Digital images of the IHC and IF staining were captured using a Leica DM750 microscope and quantification was performed using Image J. For histological quantification, every fifth 40-μm-thick section from the region between bregma − 2.69 mm and − 3.15 mm (Paxinos mouse brain atlas) was analyzed. Four to six sections per brain were stained and quantified. The number of target protein-positive cells per area (mm^2^) was counted, and the co-localization rate (%) was calculated using the following formula: {(the number of target protein-positive cells co-stained with cell type marker)/the number of cell type marker-positive cells} × 100.

### Statistical analysis

The differences between experimental groups were determined using one-way analysis of variance, and post-hoc comparisons were made using least significant difference tests. This analysis was conducted using SPSS for Windows, version 18.0 (SPSS Inc., Chicago, IL, USA). All values are reported as mean ± standard error of mean (SEM). A *p* value < 0.05 was considered statistically significant.

## Results

### PAP exhibits anti-inflammatory effects in LPS-stimulated microglial cells, mediated through PDE10 inhibition

**Fig. 1:**
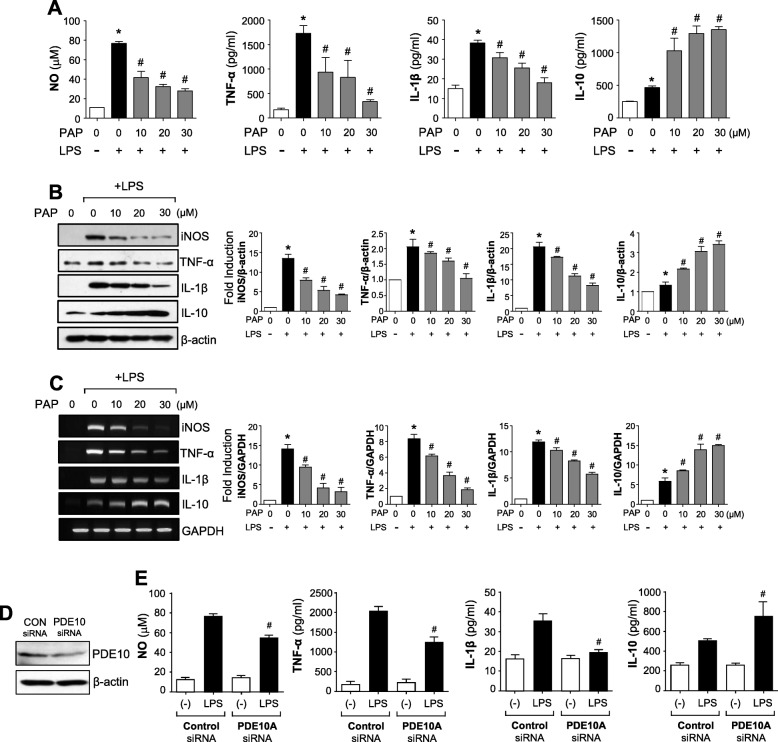
Effect of PAP on NO and inflammatory molecules in LPS-stimulated BV2 microglia. **a** BV2 cells were pretreated with PAP for 1 h and incubated with LPS (100 ng/mL). After incubation for 16 h, the supernatants were obtained and the levels of nitrite, TNF-α, IL-1β, and IL-10 were measured. **b**, **c** BV2 cells were pretreated with PAP for 1 h and incubated with LPS for 6 h. Western blot analysis (**b**) and RT-PCR (**c**) were performed to measure the expression of inflammatory molecules. Representative gels are shown in the left panel, and the quantification of three independent experiments is shown in the right panel. Data are shown as the mean ± SEM of three independent experiments. *p < 0.05 vs. control group; ^#^*p* < 0.05 vs. LPS-treated samples. **d** BV2 cells were transfected with PDE10-specific or control siRNA. Downregulation of PDE10 expression by PDE10 siRNA was confirmed by Western blot analysis. **e** The cells transfected with PDE10 siRNA or control siRNA were treated with LPS for 16 h. Then, the amounts of NO, TNF-α, IL-1β, and IL-10 released into the media were determined. ^#^*p* < 0.05 vs. control siRNA-transfected cells in the presence of LPS

To determine the anti-inflammatory role of PAP, the effect of PAP on the levels of NO and cytokines was investigated. PAP concentrations of 10, 20, and 30 μM were used for all experiments since these had no significant effect on cell viability after 24 h (data not shown). PAP significantly suppressed the production of the proinflammatory molecules NO, TNF-α, and IL-1β and increased the production of the anti-inflammatory cytokine IL-10 in LPS-stimulated BV2 cells (Fig. 1a). Data from the Western blot analysis showed that PAP reduced the protein expression of iNOS, TNF-α, and IL-1β and increased the expression of IL-10 in BV2 cells (Fig. 1b). Using RT-PCR, we found that PAP regulated the expression of iNOS and cytokines at the mRNA level (Fig. 1c). PAP also inhibited LPS-induced expression of iNOS, TNF-α, and IL-1β in primary microglia (Additional file 2: Figure S1). Since PAP is a PDE10 inhibitor, we examined whether PDE10 inhibition recapitulates the effect of PAP. PDE10 knockdown by siRNA suppressed NO, TNF-α, and IL-1β production and increased IL-10 production in LPS-stimulated BV2 cells (Fig. 1d, e), suggesting that the anti-inflammatory effects of PAP are at least partly mediated through PDE10 inhibition.

### PAP inhibits the phosphorylation of MAPKs and Akt, and the activities of NF-kB and AP-1 in LPS-stimulated BV2 microglial cells

**Fig. 2:**
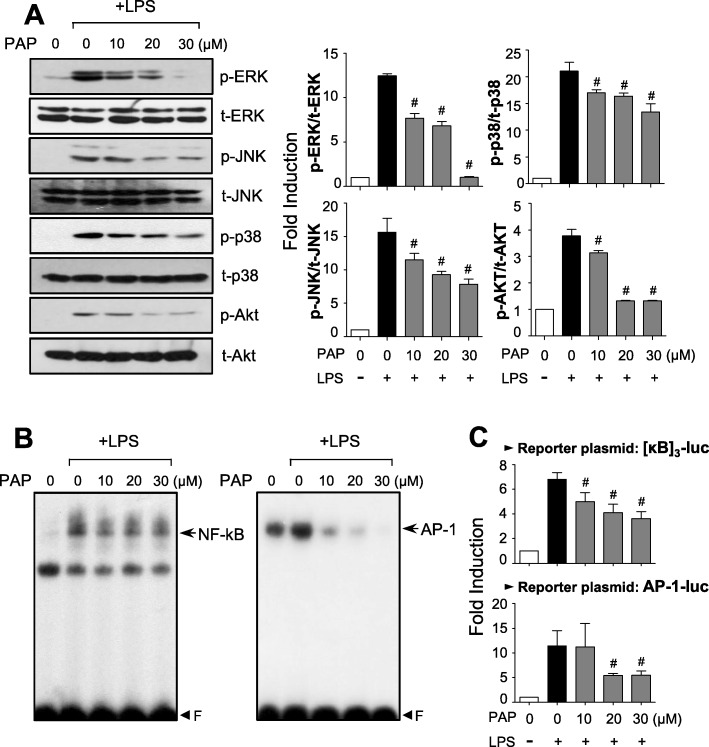
Effect of PAP on MAPKs, Akt, and NF-κB/AP-1 activities in LPS-stimulated BV2 cells. **a** Cell lysates were prepared from BV2 cells treated with LPS for 30 min in the absence or presence of PAP, and Western blotting was performed to determine the effect of PAP on MAPKs and Akt activity. Quantification data are shown in the right panels (*n* = 3). Levels of the phosphorylated forms of MAPKs and Akt were normalized to the total forms and expressed as fold changes vs. untreated control samples, which were arbitrarily set to 1. **b** EMSA for NF-κB and AP-1 was performed using nuclear extracts prepared from BV2 cells treated with PAP in the presence of LPS for 3 h. **c** Transient transfection analysis of [κB]_3_-luc, and AP-1-luc reporter gene activity. Data are shown as the mean ± SEM of three independent experiments. ^#^*p* < 0.05 vs. LPS-treated samples

We also examined the effect of PAP on MAPKs and the PI3K/Akt signaling pathway, known to play an important role in proinflammatory gene expression by modulating transcription factors such as NF-κB and AP-1 [41, 42]. PAP inhibited LPS-induced phosphorylation of MAPKs and Akt in BV2 cells (Fig. 2a). The DNA binding activity and reporter gene activity of both NF-κB and AP-1 were also inhibited by PAP in LPS-stimulated BV2 cells (Fig. 2b, c), suggesting that PAP regulates inflammatory responses partly by modulating PI3K/Akt, MAPKs, NF-κB, and AP-1 signaling pathways.

### PAP inhibits ROS production by suppressing the mRNA expression and phosphorylation of p47phox, a component of NADPH oxidases, and enhancing the Nrf2/ARE signaling pathway

**Fig. 3:**
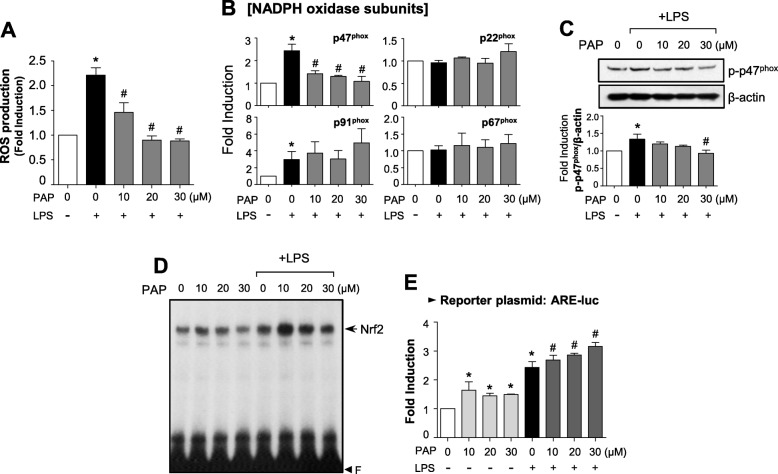
PAP reduced ROS production via suppression of a NADPH oxidase subunit and upregulation of Nrf2/ARE signaling. **a** BV2 cells were treated with PAP 1 h prior to LPS stimulation for 16 h, and intracellular ROS level was measured by the DCF-DA method. **b** RT-PCR to assess mRNA expression of NADPH oxidase subunits (p47^phox^, gp91^phox^, p67^phox^, and p22^phox^) in BV2 cells. **c** Western blot analysis to assess phosphorylation of the p47^phox^ subunit. BV2 cells were treated with PAP 1 h followed by LPS (100 ng/mL, 30 min) and then subjected to immunoblot analysis using antibodies against phospho-p47^phox^. **d** EMSA to assess Nrf2 DNA binding activity. **e** Transient transfection analysis of ARE-luc reporter gene activity. Data are shown as the mean ± SEM of three independent experiments. *p < 0.05 vs. control group; ^#^*p* < 0.05 vs. LPS-treated group

Reactive oxygen species (ROS) are known to be early signaling inducers in inflammatory responses [43]. Thus, we investigated the effect of PAP on ROS production in LPS-stimulated BV2 cells. DCF-DA assay data showed that PAP inhibited the production of intracellular ROS induced by LPS (Fig. 3a). Since NADPH oxidases are major enzymes involved in ROS generation, we investigated whether PAP influences the components of these enzymes. As shown in Fig. 3b, PAP suppressed the mRNA expression of p47phox, but not other components, in LPS-stimulated BV2 cells. In addition, PAP inhibited LPS-induced phosphorylation of p47phox (Fig. 3c). Next, we examined the effect of PAP on antioxidant Nrf2/ARE signaling. EMSA data showed that PAP enhanced LPS-induced Nrf2 binding to ARE (Fig. 3d). Moreover, PAP induced ARE-driven luciferase activity in the absence and presence of LPS (Fig. 3e).

### The PKA/CREB pathway plays a crucial role in the anti-inflammatory effect of PAP

**Fig. 4:**
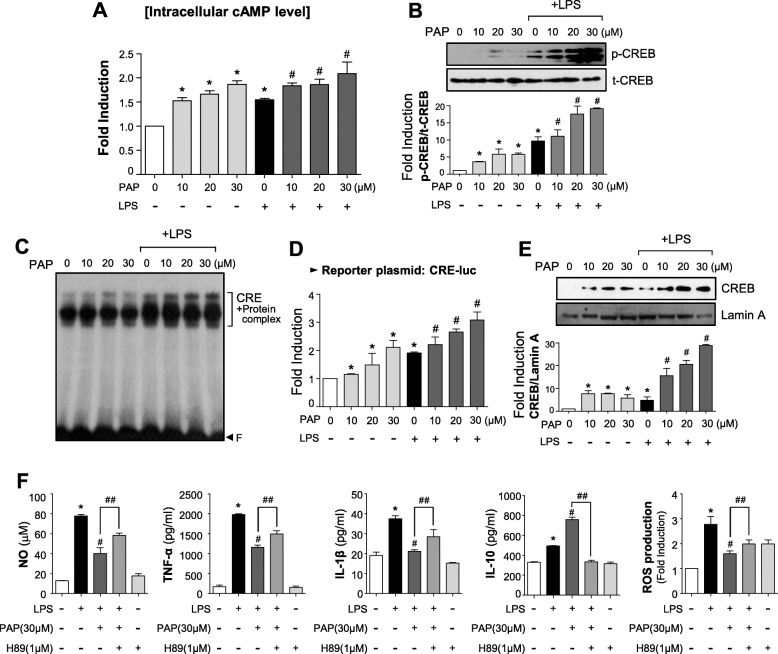
PAP enhanced PKA/CREB signaling, and H89 reversed the anti-inflammatory effects of PAP in LPS-stimulated BV2 cells. **a** Intracellular cAMP level was measured in BV2 cells treated with PAP in the presence or absence of LPS for 30 min. **b** Western blot analysis to assess the phosphorylated and total forms of CREB using the same cell lysates as **a**. **c** EMSA to assess CREB DNA binding activity. **d** Transient transfection analysis of CRE-luc reporter gene activity. **e** Western blot to detect the nuclear translocation of CREB. **f** Cells were pretreated with H89 for 30 min, then treated with PAP for 1 h followed by LPS for 16 h. The production of NO, TNF-α, IL-1β, IL-10, and ROS was measured. Data are shown as the mean ± SEM of three independent experiments. *p < 0.05, control vs. LPS-treated group; ^#^*p* < 0.05, LPS vs. LPS+PAP-treated group; ^##^*p* < 0.05, LPS+PAP vs. LPS+PAP+H89 group

We investigated whether PAP affects intracellular cAMP level by modulating PDE. As expected, PAP increased cAMP level in BV2 cells in the presence and absence of LPS (Fig. 4a). PAP also increased CREB phosphorylation (Fig. 4b) and other CREB activities such as DNA binding, transcriptional activity, and nuclear translocation (Fig. 4c–e). To determine whether the PKA/CREB pathway is involved in the anti-inflammatory mechanism of PAP, BV2 cells were pre-treated with a PKA inhibitor, H89, before PAP and LPS stimulation. The data showed that H89 significantly attenuated PAP-mediated suppression of NO, TNF-α, IL-1β, and ROS and attenuated PAP-induced IL-10 production (Fig. 4f). Overall, these results indicate that the PKA pathway plays a pivotal role in the anti-inflammatory/antioxidant mechanisms of PAP.

### PAP increases PPARγ and Nrf2/ARE signaling, in which PKA is an upstream modulator

**Fig. 5:**
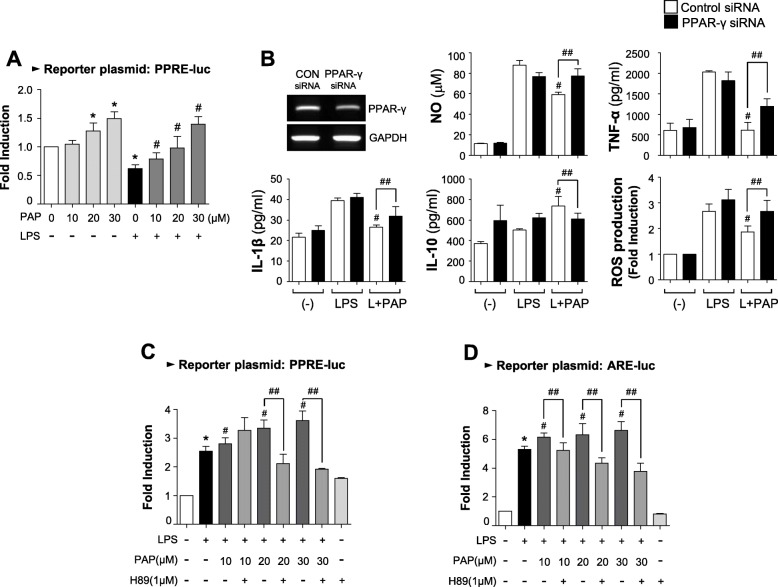
PAP increased PPARγ activity, which also depends on PKA signaling. **a** Transient transfection analysis of PPRE-luc reporter gene activity. **b** BV2 cells were transfected with PPAR-γ-specific or control siRNA, and treated with LPS in the absence or presence of PAP (30 μM) for 16 h. Then, the production of NO, ROS, and cytokines was measured. ^#^*p* < 0.05 vs. LPS-treated samples; ^##^*p* < 0.05 vs. control siRNA-transfected cells in the presence of LPS+PAP. **c**, **d** BV2 cells transfected with the reporter plasmid (ARE-luc, and PPRE-luc) were pretreated with H89 for 30 min. They were then treated by PAP for 1 h followed by LPS for 6 h. A luciferase assay was performed to measure the reporter gene activities of PPRE (**c**) and ARE (**d**). Data are shown as the mean ± SEM of three independent experiments. *p < 0.05, control vs. LPS-treated group; ^#^*p* < 0.05, LPS vs. LPS+PAP-treated group; ^##^*p* < 0.05, LPS+PAP vs. LPS+PAP+H89 group

Next, we examined the effect of PAP on PPARγ, a key transcription factor for the resolution of inflammation [44]. PAP increased the transcriptional activity of PPARγ, as shown by the cell-based reporter gene assay in BV2 cells (Fig. 5a). To determine whether PPARγ has an effect on the anti-inflammatory role of PAP, a siRNA knockdown experiment was performed. Data from the PPARγ siRNA transfection showed that the anti-inflammatory effect of PAP is partially reversed by PPARγ knockdown (Fig. 5b), suggesting that PPARγ plays an important role in the action of PAP. To investigate whether the upregulation of PPARγ is related to PKA signaling, BV2 cells were treated with H89 before LPS/PAP treatment and a PPRE-luc reporter gene assay was performed. As shown in Fig. 5c, H89 attenuated the effect of PAP on PPRE-luc activity. Moreover, H89 reversed the effect of PAP on ARE-luc activity (Fig. 5d). These data suggest that PKA plays a role as an upstream modulator of PPARγ and Nrf2/ARE signaling in LPS/PAP-treated BV2 cells.

### PAP inhibits microglial activation and proinflammatory gene expression, which is reversed by the PKA inhibitor H89, in the brains of LPS-injected mice

**Fig. 6:**
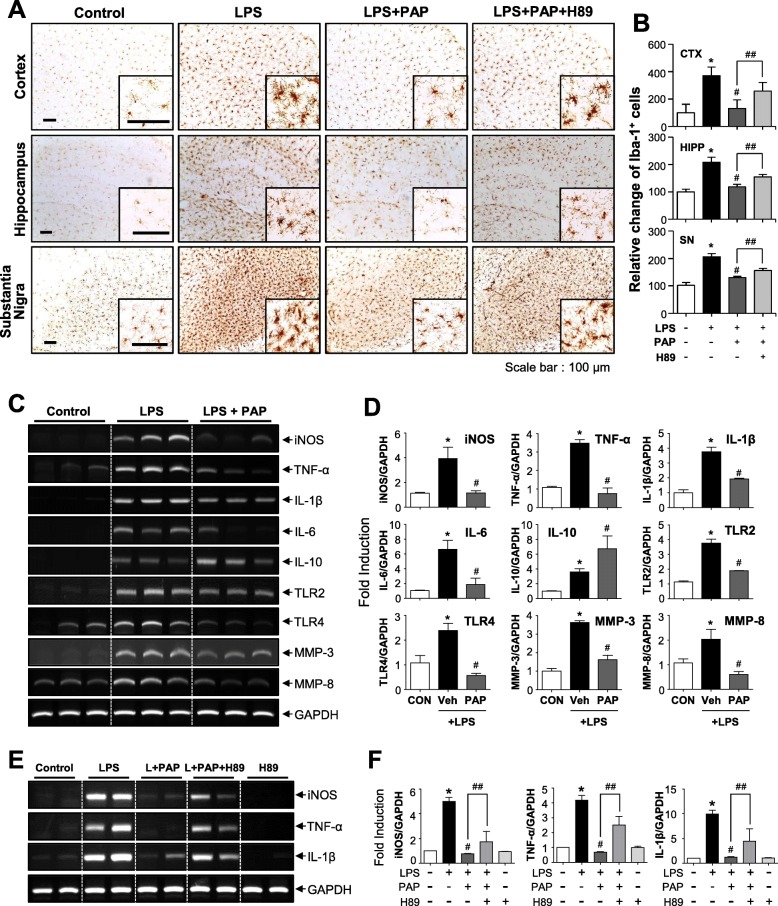
Effects of PAP on microglial activation and the mRNA expression of inflammatory markers in the brains of LPS-injected mice. **a**, **b** Immunohistochemical staining for Iba-1 and quantification of the number of Iba-1-positive microglia 3 days after LPS injection (each group *n* = 4–5). Microglial activation in the cortex, hippocampus, and substantia nigra of LPS-injected mice was reduced by PAP (30 mg/kg), and this was reversed by H89 treatment. Representative images (**a**) and the quantification of data (**b**) are shown. Scale bars, 100 μm. **c**, **d** Effects of PAP on the mRNA levels of iNOS, cytokines, microglial activation markers (TLR2, TLR4), and proinflammatory MMPs (MMP-3, MMP-8) in the cortices of LPS-injected mice (each group *n* = 4). Representative gels (**c**) and quantification data (**d**) are shown. **e**, **f** Effect of H89 on PAP-mediated suppression of proinflammatory gene expression in LPS-injected mouse brains. *p < 0.05, control vs. LPS-treated group; ^#^*p* < 0.05, LPS vs. LPS+PAP-treated group; ^##^*p* < 0.05, LPS+PAP vs. LPS+PAP+H89 group

To verify the effects of PAP in vivo, PAP was injected into mice prior to LPS administration. After 3 days of LPS injection, microglial activation and the expression of proinflammatory molecules were evaluated in LPS-injected mouse brains. Systemic LPS increased the number of Iba-1-positive cells in the cortex, hippocampus, and substantia nigra. PAP decreased the number of these cells, indicating that this suppressed microglial activation (Fig. 6a, b). Moreover, PAP suppressed the mRNA expression of iNOS, proinflammatory cytokines, matrix metalloproteinases (MMPs; MMP-3, MMP-8), and toll-like receptors (TLRs; TLR2, TLR4) and increased the expression of the anti-inflammatory cytokine IL-10 in LPS-injected mouse brains (Fig. 6c, d). Next, to investigate whether PKA signaling is also involved in the anti-inflammatory effect of PAP in vivo, H89 was injected 1 h before every PAP injection. As shown in Fig. 6 a and e, H89 treatment reversed the PAP-mediated suppression of microglial activation and iNOS, TNF-α, and IL-1β expression. These results indicate that the PKA pathway is also involved in the anti-inflammatory action of PAP in LPS-injected mouse brains.

### PAP exerts anti-inflammatory and neuroprotective effects in MPTP-induced PD model mice

**Fig. 7:**
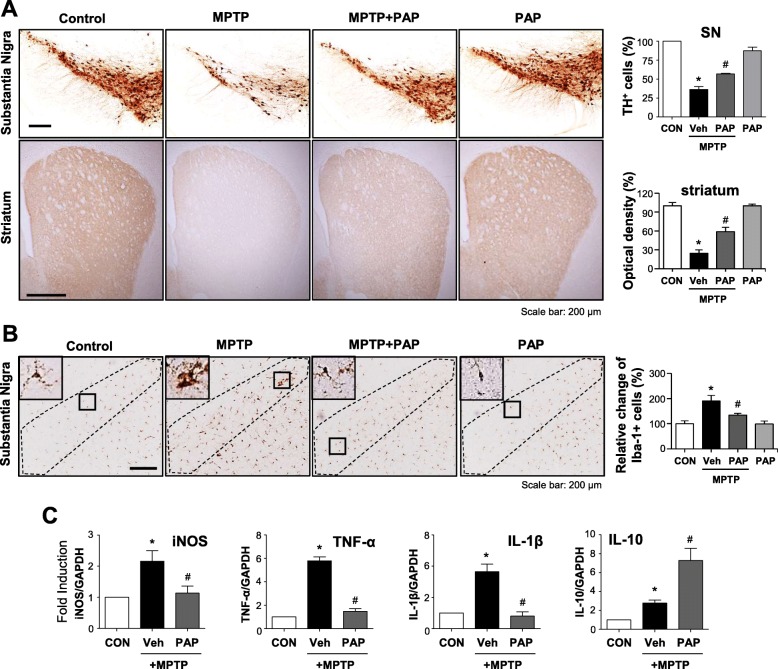
Effect of PAP on dopaminergic neuronal cell death, microglial activation, and the expression of inflammatory genes in the brains of MPTP-injected mice. **a** Representative images of TH-positive neuronal cells in the substantia nigra and striatum (each group *n* = 4–5). Quantitative analysis was performed by measuring the number of TH-positive cells in the substantia nigra, and the optical density of TH-positive fibers in the striatum (right panel). **b** Representative images of Iba-1-positive microglial cells in the substantia nigra (each group *n* = 4–5). **c** Real-time PCR analysis was performed to examine the expression of iNOS and cytokines in the substantia nigra (each group *n* = 4). *p < 0.05 vs. control group; ^#^*p* < 0.05 vs. MPTP group

We investigated whether PAP has anti-inflammatory and neuroprotective effects in MPTP-induced PD model mice. An injection of PAP was administered once a day for 3 days before MPTP injection, and mice were sacrificed 7 days later for analysis to be conducted. Dopaminergic neuronal cell death was measured by tyrosine hydroxylase (TH) staining. The data showed that PAP recovered MPTP-mediated neuronal cell death in the substantia nigra and striatum (Fig. 7a; SN, F_3, 12_ = 49.25, *p* < 0.01; striatum, F_3, 12_ = 49.24, *p* < 0.01). Moreover, PAP exerted anti-inflammatory effects by reducing MPTP-induced microglial activation (Fig. 7b; F_3, 12_ = 9.33, *p* < 0.01), and the mRNA levels of proinflammatory cytokines and enhancing the mRNA level of the anti-inflammatory cytokine IL-10 (Fig. 7)c. Representative high-magnification images of microglia are shown in Additional file 2: Figure S2.

### The anti-inflammatory and neuroprotective effects of PAP are dependent on the PKA/CREB signaling pathway in MPTP-induced PD model mice

**Fig. 8:**
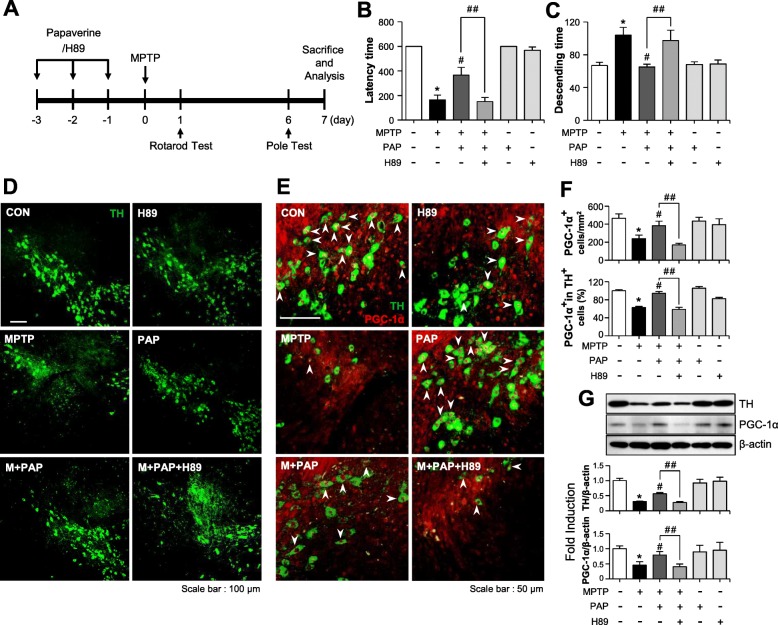
Effect of PAP/H89 on locomotor activity and the expression of TH and PGC-1α in the brains of MPTP-injected mice. **a** A schematic of the experimental procedure. Mice were injected with PAP (30 mg/kg, i.p.) every day for 3 days before MPTP injection. H89 was injected (1 mg/kg, i.p.) 1 h prior to every PAP injection. Mice were sacrificed 7 days following MPTP injection, and histological and biochemical analyses were performed. **b**, **c** Rotarod and pole tests were performed 1 and 6 days after MPTP injection, respectively (each group *n* = 12–14). **d**, **e** Immunostaining results showing TH and PGC-1α expression in the substantia nigra (each group *n* = 6–7). The white arrows indicate TH-positive cells with PGC-1α in their nucleus. **f** Quantification of TH and/or PGC-1α- positive cells in the substantia nigra pars compacta. **g** The protein extracts from the substantia nigra of each group were subjected to Western blot analysis using TH or PGC-1α antibodies (each group *n* = 5). Representative blots are provided in the upper panel, and quantification of the Western blot data is shown in bottom panel. *p < 0.05, control vs. MPTP-treated group; ^#^*p* < 0.05, MPTP vs. MPTP+PAP-treated group; ^##^*p* < 0.05, MPTP+PAP vs. MPTP+PAP+H89 group

**Fig. 9:**
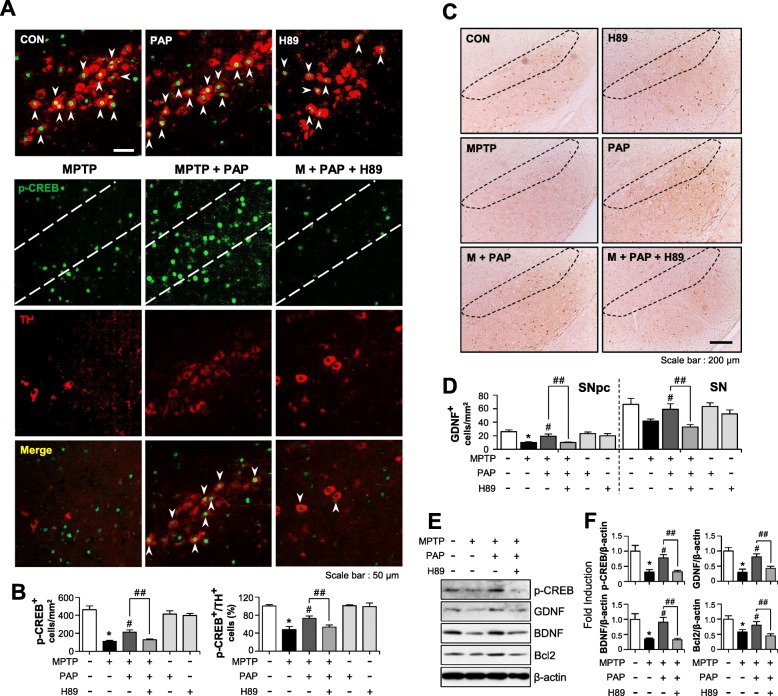
Effect of PAP/H89 on p-CREB level and its downstream neuroprotective factors in the brains of MPTP-injected mice. **a** Immunostaining results showing TH and p-CREB expression in the substantia nigra (each group *n* = 6–7). The white arrows indicate cells positive for both TH and p-CREB. **b** Quantification of p-CREB and/or TH-positive cells in the substantia nigra. **c**, **d** IHC results for GDNF staining in the substantia nigra (**c**), and quantification data from the substantia nigra pars compacta and substantia nigra region (each group *n* = 6–7). **e** The protein extracts from the substantia nigra of each group were subjected to Western blot analysis using p-CREB, GDNF, Bcl2, and BDNF antibodies (each group *n* = 5), and representative blots are provided. **f** Quantification of Western blot data. *p < 0.05, control vs. MPTP-treated group; ^#^*p* < 0.05, MPTP vs. MPTP+PAP-treated group; ^##^*p* < 0.05, MPTP+PAP vs. MPTP+PAP+H89 group

**Fig. 10:**
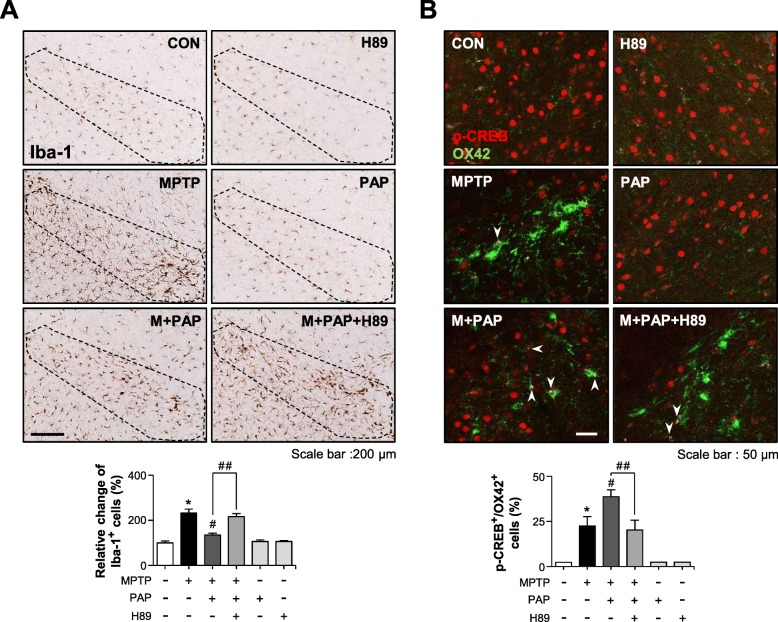
Effect of H89 on PAP-mediated microglial inactivation and CREB phosphorylation in the brains of MPTP-injected mice. **a** Immunohistochemical staining for Iba-1 and quantification of the number of Iba-1-positive microglia in the substantia nigra of MPTP-injected mice (each group *n* = 6–7). **b** The p-CREB levels in microglia were measured by immunofluorescence staining. The white arrows indicate cells positive for both OX42 and p-CREB. *p < 0.05, control vs. MPTP-treated group; ^#^*p* < 0.05, MPTP vs. MPTP+PAP-treated group; ^##^*p* < 0.05, MPTP+PAP vs. MPTP+PAP+H89 group

To investigate whether the PKA pathway also governs the anti-inflammatory and neuroprotective effects of PAP in MPTP-injected mice, mice were administered H89 before PAP injection, and behavioral, immunohistochemical, and biochemical analyses were then performed (Fig. 8a). The data from the rotarod and pole tests showed that MPTP injection impaired locomotor activity, and PAP improved this. H89 reversed this effect of PAP (Fig. 8b, c; rotarod, F_5, 67_ = 30.40, *p* < 0.05; pole, F_5, 62_ = 5.95, *p* < 0.05). In addition, H89 attenuated PAP-mediated neuroprotection and PGC-1α translocation into dopaminergic neurons (Fig. 8d, e; PGC-1α, F_5, 37_ = 13.76, *p* < 0.01; PGC-1α+TH, F_5, 37_ = 4.15, *p* < 0.01). In accordance with these data, Western blot analysis showed that MPTP injection reduced the levels of TH and PGC-1α, and this was recovered by PAP. Once again, the effects of PAP were reversed by H89 treatment (Fig. 8f, g). Next, we examined the effect of PAP and H89 on CREB phosphorylation and its downstream neurotrophic factors BDNF, GDNF, and Bcl2 by IHC and Western blot analyses. MPTP injection led to a reduction in the protein levels of p-CREB, BDNF, GDNF, and Bcl2 in the substantia nigra, and these were all recovered by PAP treatment (Fig. 9; pCREB, F_5, 18_ = 31.84, *p* < 0.01; pCREB+TH, F_5, 18_ = 30.35, *p* < 0.01; GDNF SNpc, F_5, 36_ = 7.69, *p* < 0.01; GDNF SN, F_5, 36_ = 4.50, *p* < 0.01). However, H89 treatment attenuated the effects of PAP. High-magnification images of GDNF-positive cells in the SN are shown in Additional file 2: Figure S3. Finally, we examined the effect of H89 on microglial activation in the brains of MPTP/PAP-injected mice. As shown in Fig. 10, H89 also reversed PAP-mediated microglial inactivation (F_5, 37_ = 29.20, *p* < 0.01) and CREB phosphorylation in microglia (F_5, 18_ = 22.20, *p* < 0.01). The data collectively support the critical role of PKA in the neuroprotective and anti-inflammatory functions of PAP in the brains of PD model mice.

## Discussion

In the first part of this study, we demonstrated the anti-inflammatory effects of PAP in LPS-stimulated BV2 microglia. PAP exerts anti-inflammatory effects by suppressing proinflammatory molecules such as NO, proinflammatory cytokines, and ROS and enhancing the anti-inflammatory cytokine IL-10. These anti-inflammatory effects are mediated via the downregulation of MAPKs, PI3K/Akt, and NF-κB and the upregulation of PKA, Nrf2/ARE, and PPARγ signaling pathways. Blocking PKA signaling reverses the action of PAP, suggesting that the PKA pathway plays a major role in the anti-inflammatory mechanisms of PAP in LPS-stimulated microglia. In the second part of this study, we demonstrated the anti-inflammatory and neuroprotective effects of PAP in LPS- or MPTP-injected mouse model. PAP exerts anti-inflammatory effects by inhibiting microglial activation and the expression of proinflammatory mediators in LPS-injected mouse brains. In addition, PAP exerts neuroprotective effects by recovering the neuronal cell death and impaired locomotor activity induced by MPTP in mice. PAP also exerts anti-inflammatory effects in MPTP-injected mice by suppressing microglial activation and the expression of proinflammatory cytokines. Finally, the effect of the PKA inhibitor H89 on the anti-inflammatory and neuroprotective effects of PAP indicates the importance of the PKA pathway in mediating these effects.

PD is a neurodegenerative disease characterized by the preferential death of the dopaminergic neurons in the substantia nigra innervating the striatum. The symptoms of this disease include resting tremor, rigidity, bradykinesia, and postural instability [45]. There currently exists no cure for PD, but various therapies are utilized to improve symptoms, namely pharmacological agents, surgery, and physical exercise. Currently, PDE inhibitors are undergoing clinical trials for neurodegenerative and neuropsychiatric disorders such as HD, AD, schizophrenia, and depression [46]. However, no clinical trials are currently exploring the use of a PDE inhibitor for PD. There exist eleven families of PDE, one of which is PDE10A. This is particularly expressed in the medium spiny neurons of the striatum, and acts to integrate dopaminergic and glutamatergic signal transduction by modulating cAMP/cGMP levels. PDE10A helps to regulate synaptic transmission, neuronal excitability, and synaptic plasticity [37]. Therefore, PDE10 has been suggested as a logical target for the correction of striatal dopamine receptor signaling, which is known to be deficient in PD [46, 47]. In the present study, we found that the selective PDE10 inhibitor PAP rescued impaired movement in MPTP-injected PD mice, demonstrated using the rotarod and pole tests. Moreover, PAP increased the expression of neuronal survival factors such as BDNF, GDNF, Bcl2, and PGC-1α that are under the control of PKA/CREB signaling, thereby promoting the survivability of dopaminergic neurons in the substantia nigra. Previous studies have reported that PAP induces reversible opening of the BBB and mediates transient BBB permeability [48, 49]. These findings therefore suggest that PAP may be a potential therapeutic agent for PD.

Previous studies have reported that the PKA/CREB pathway contributes to the resolution of inflammation and ROS detoxification [50–52]. This anti-inflammatory effect is closely related to CREB-mediated upregulation of the anti-inflammatory cytokine IL-10 and inhibition of proinflammatory cytokines such as TNF-α [53, 54]. CREB also induces the expression of PGC-1α, a key effector of ROS detoxifying enzyme expression and mitochondrial biogenesis [23]. Furthermore, we previously demonstrated that the PKA/CREB pathway governs the expression of hemeoxygenase-1 in microglial cells [52]. In addition to CREB, the present study found that PAP also increased the activities of Nrf2 and PPAR-γ, which have anti-inflammatory/antioxidant functions by inhibiting NF-κB and ROS production in microglia [38, 44, 55]. Interestingly, PKA inhibition by H89 reversed this, suggesting that PKA is an upstream modulator of Nrf2 and PPARγ. Our data collectively suggest that PKA is the master regulator of the anti-inflammatory and antioxidant functions of PAP in activated microglia.

As a potent PDE10 inhibitor, PAP activates cAMP/PKA signaling in striatopallidal and striatonigral medium spiny neurons. This leads to the inhibition of dopamine D2 receptor signaling in striatopallidal neurons, and the potentiation of dopamine D1 receptor signaling in striatonigral neurons. Therefore, PDE10 inhibition by PAP has been suggested as a therapeutic target for neuropsychotic disorders such as schizophrenia [27, 56]. Moreover, PAP improved cognitive impairment in a R6/1 HD mouse model, partly by increasing hippocampal pGluA1 and p-CREB levels, suggesting a potential role in HD treatment [30]. However, the therapeutic effect of PAP in PD models has not been reported until now. In the present study, we demonstrated that PAP suppressed microglial activation and recovered dopaminergic neuronal cell death via PKA signaling in sepsis and PD model mice. Using siRNA knockdown experiments, we demonstrated that the anti-neuroinflammatory effects of PAP are mediated through PDE10. In support of this, we found that MP-10 (PF-02545920), a highly selective and potent PDE10 inhibitor, recapitulates the effect of PAP in LPS-stimulated microglia and septic mouse brains (data not shown). However, we cannot exclude the possibility that a PDE10-independent mechanism is also involved in the anti-inflammatory and neuroprotective effects of PAP. Further study is therefore warranted to address this issue.

## Conclusions

The present study reports, for the first time, the anti-inflammatory and neuroprotective effects of PAP in systemic inflammation and PD mouse models. We demonstrated that the PKA signaling pathway plays a key role in the anti-inflammatory and neuroprotective mechanisms of PAP by modulating various downstream effectors. Therefore, PAP may be a promising therapeutic candidate for neuroinflammatory and neurodegenerative disorders such as PD.

## Supplementary information

**Additional file 1: Table S1.** List of primary antibodies used in Western blot analysis. **Table S2**. List of primary antibodies used in immunohistochemical and immunofluorescence staining

**Additional file 2: Figure S1.** Effect of PAP on the expression of iNOS, TNF-α, and IL-1β in LPS-stimulated rat primary microglia. **Figure S2.** Morphological changes of microglia in response to MPTP. **Figure S3.** High magnification images of GDNF-positive cells in the substantia nigra of MPTP-injected mice

## Data Availability

The datasets used and/or analyzed during the current study are available from the corresponding author on reasonable request.

## Abbreviations

AP-1,: Activator protein-1
ARE,: Antioxidant response element
BBB,: Blood-brain barrier
BDNF,: Brain-derived neurotrophic factor
cAMP,: Adenosine 3′,5′-cyclic monophosphate
CREB,: cAMP response element-binding protein
EMSA,: Electrophoretic mobility shift assay
ERK,: Extracellular signal-regulated kinase
GDNF,: Glial cell line-derived neurotrophic factor
HD,: Huntington’s disease
IF,: Immunofluorescence
IHC,: Immunohistochemical
IL,: Interleukin
iNOS,: Inducible nitric oxide synthase
LPS,: Lipopolysaccharide
MAPK,: Mitogen-activated protein kinase
MMP,: Matrix metalloproteinase
MPTP,: 1-Methyl-1,2,3,6-tetrahydropyridine
NF-κB,: Nuclear factor-κB
NO,: Nitric oxide
Nrf2,: Nuclear factor-erythroid 2 (NF-E2)–related factor 2
PAP,: Papaverine
PD,: Parkinson’s disease
PDE,: Phosphodiesterase
PI3K,: Phosphoinositol 3-kinase
PKA,: Protein kinase A
PPAR,: Peroxisome proliferator-activated receptor
ROS,: Reactive oxygen species
siRNA,: Small interference RNA
SN,: Substantia nigra
TH,: Tyrosine hydroxylase
TLR,: Toll-like receptor
TNF,: Tumor necrosis factor
