# Supplementary material for: The phosphodiesterase 10 inhibitor papaverine exerts anti-inflammatory and neuroprotective effects via the PKA signaling pathway in neuroinflammation and Parkinson’s disease mouse models
Source: J Neuroinflammation. 2019 Dec 2;16:246. doi: 10.1186/s12974-019-1649-3 (PMC6888927; doi:10.1186/s12974-019-1649-3)
Supplement: Supplementary file 1 — Additional file 1: Table S1. List of primary antibodies used in Western blot analysis. Table S2. List of primary antibodies used in immunohistochemical and immunofluorescence staining [file 12974_2019_1649_MOESM1_ESM.pdf]

Supplementary Table 1. List of primary antibodies used in western blot analysis

Antigen	Manufacturer	Catalog number	Dilution
β-actin	Sigma	A1978	1:10000
Akt	Cell signaling	9272	1:2000
p-Akt	Cell signaling	9271	1:2000
Bcl2	Abcam	ab59348	1:3000
BDNF	Sigma	SAB2108004	1:2000
CREB	Cell signaling	9197	1:2000
p-CREB	Cell signaling	9198	1:1000
ERK	Cell signaling	9102	1:2000
p-ERK	Cell signaling	9101	1:2000
GDNF	Abcam	ab18956	1:1000
Iba-1	Wako	019-19741	1:1000
IL-1β	R&D	AF-401-NA	1:1000
IL-10	Abcam	9969	1:1000
iNOS	BD bioscience	610431	1:1000
JNK	Cell signaling	9252	1:2000
p-JNK	Cell signaling	9251	1:2000
Lamin A	Santa Cruz	20680	1:1000
PDE10A	Santa Cruz	515023	1:1000
PGC-1α	Millipore	ST1202	1:1000
p38	Cell signaling	9212	1:2000
p-p38	Cell signaling	9211	1:2000
p-p47 phox	Assaybiotech	A8391	1:1000
TH	Santa Cruz	14007	1:2000
TNF-α	Santa Cruz	1350	1:500

Supplementary Table 2. List of primary antibodies used in immunohistochemical and immunofluorescence staining

Antigen	Manufacturer	Catalog number	Dilution
GDNF	Abcam	ab18956	1:300
CD11b (OX-42)	Santa Cruz	28664	1:500
p-CREB	Cell signaling	9198	1:2000
Iba-1	Wako	019-19741	1:500
PGC-1α	Millipore	ST1202	1:2000
TH	Santa Cruz	14007	1:1000
