# Supplementary material for: The phosphodiesterase 10 inhibitor papaverine exerts anti-inflammatory and neuroprotective effects via the PKA signaling pathway in neuroinflammation and Parkinson’s disease mouse models
Source: J Neuroinflammation. 2019 Dec 2;16:246. doi: 10.1186/s12974-019-1649-3 (PMC6888927; doi:10.1186/s12974-019-1649-3)
Supplement: Supplementary file 2 — Additional file 2: Figure S1. Effect of PAP on the expression of iNOS, TNF-α, and IL-1β in LPS-stimulated rat primary microglia. Figure S2. Morphological changes of microglia in response to MPTP. Figure S3. High magnification images of GDNF-positive cells in the substantia nigra of MPTP-injected mice [file 12974_2019_1649_MOESM2_ESM.pdf]

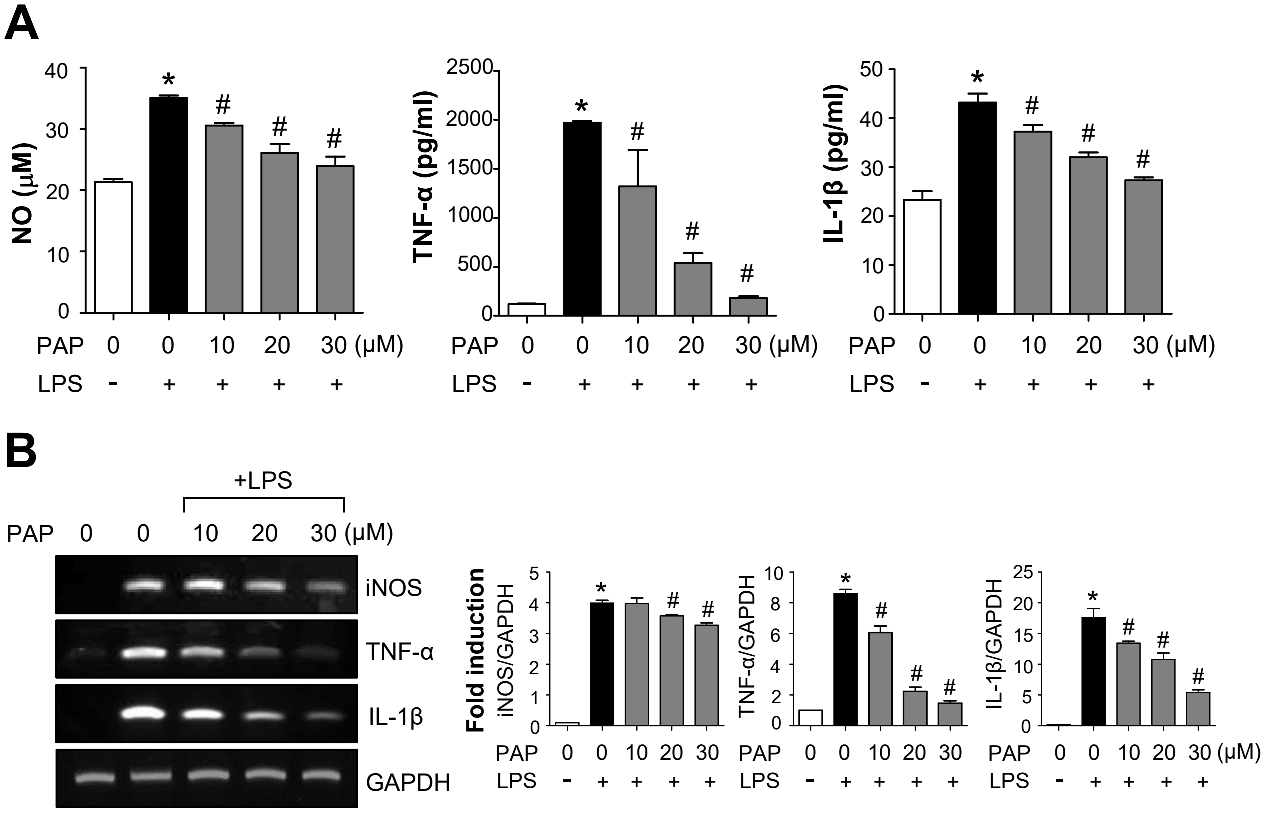

Figure S1: **Effect of PAP on the expression of iNOS, TNF-α, and IL-1β in LPS-stimulated rat primary microglia.** (A) Primary microglia were pretreated with PAP for 1 h and incubated with LPS (10 ng/ml). After incubation for 16 h, the supernatants were obtained and the levels of nitrite, TNF-α, and IL-1β, were measured. (B) Primary microglia were pretreated with PAP for 1 h and incubated with LPS for 6 h. RT-PCR were performed to measure the expression of inflammatory molecules. Representative gels are shown in the left panel, and the quantification of three independent experiments is shown in the right panel. Data are shown as the mean ± SEM of three independent experiments. **P* < 0.05 vs. control group; $^{\#}P < 0.05$ vs. LPS-treated samples.

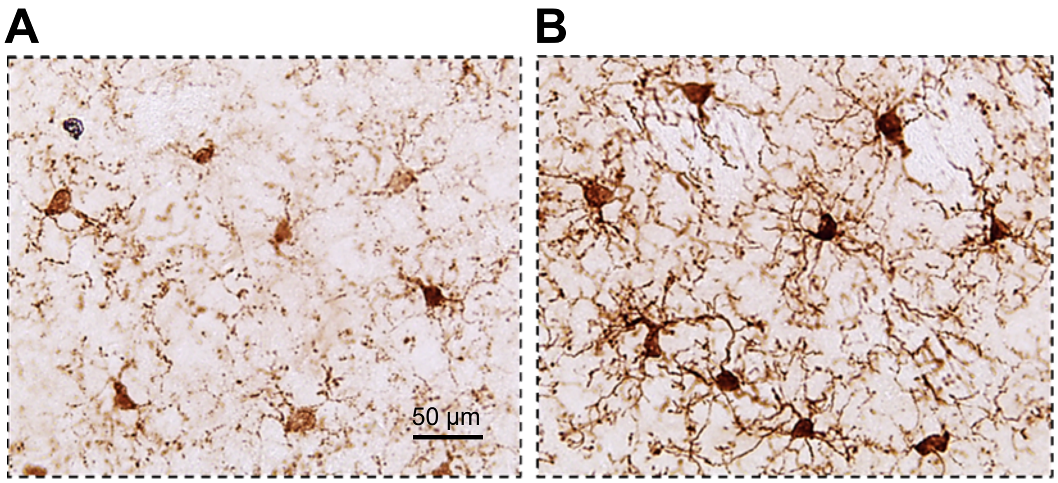

Figure S2: **Morphological changes of microglia in response to MPTP.** (A, B**)** Representative high magnification images of microglia in the substantia nigra region of MPTP-injected mice are presented. Resting form (A), Activated form (B) of Iba-1-positive microglia.

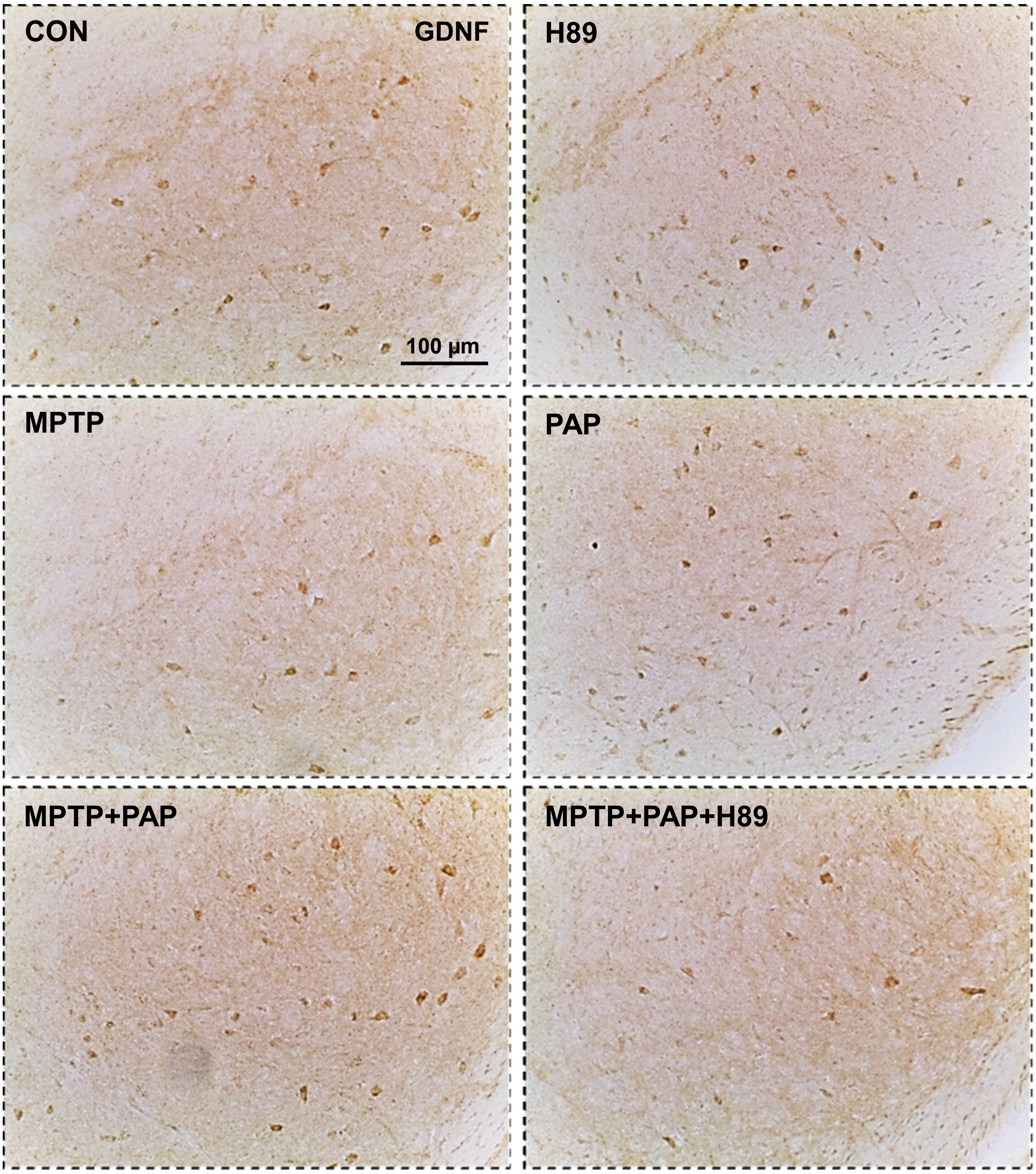

Figure S3: **High magnification images of GDNF-positive cells in the substantia nigra of MPTP-injected mice.** PAP treatment recovered MPTP-induced nigral decrease of GDNF-positive cells, which was reversed by H89 treatment.
